# Quantitative Tracking of *Salmonella* Enteritidis Transmission Routes Using Barcode-Tagged Isogenic Strains in Chickens: Proof-of-Concept Study

**DOI:** 10.3389/fvets.2017.00015

**Published:** 2017-02-14

**Authors:** Yichao Yang, Steven C. Ricke, Guillermo Tellez, Young Min Kwon

**Affiliations:** ^1^Department of Poultry Science, University of Arkansas, Fayetteville, AR, USA; ^2^Cell and Molecular Biology Program, University of Arkansas, Fayetteville, AR, USA; ^3^Department of Food Science, University of Arkansas, Fayetteville, AR, USA; ^4^Center of Food Safety, University of Arkansas, Fayetteville, AR, USA

**Keywords:** *Salmonella* Enteritidis, transmission, chickens, barcode-tagged isogenic strains, quantitative tracking

## Abstract

*Salmonella* is an important foodborne bacterial pathogen, however, a fundamental understanding on *Salmonella* transmission routes within a poultry flock remains unclear. In this study, a series of barcode-tagged strains were constructed by inserting six random nucleotides into a functionally neutral region on the chromosome of *S*. Enteritidis as a tool for quantitative tracking of *Salmonella* transmission in chickens. Six distinct barcode-tagged strains were used for infection or contamination at either low dose (10^3^ CFUs; three strains) or high dose (10^5^ CFUs; three strains) in three independent experiments (Experiment 1 oral gavage; Experiment 2 contaminated feed; Experiment 3 contaminated water). For all chick experiments, cecal and foot-wash samples were collected from a subset of the chickens at days 7 or/and 14, from which genomic DNA was extracted and used to amplify the barcode regions. After the resulting PCR amplicons were pooled and analyzed by MiSeq sequencing, a total of approximately 1.5 million reads containing the barcode sequences were analyzed to determine the relative frequency of every barcode-tagged strain in each sample. In Experiment 1, the high dose of oral infection was correlated with greater dominance of the strains in the ceca of the respective seeder chickens and also in the contact chickens yet at lesser degrees. When chicks were exposed to contaminated feed (Experiment 2) or water (Experiment 3), there were no clear patterns of the barcode-tagged strains in relation to the dosage, except that the strains introduced at low dose required a longer time to colonize the ceca with contaminated feed. Most foot-wash samples contained only one to three strains for the majority of the samples, suggesting potential existence of an unknown mechanism(s) for strain exclusion. These results demonstrated the proof of concept of using barcode tagged to investigate transmission dynamics of *Salmonella* in chickens in a quantitative manner.

## Introduction

*Salmonella* species induce bacterial illness and are also one of the leading causes of hospitalization among all the foodborne bacterial pathogens (1, 2). According to the Centers for Disease Control and Prevention, there are approximately 1.2–4 million human *Salmonella* infections in the United States each year (3–5). There are multiple sources of *Salmonella* infection in humans such as consumption of contaminated food and water or contact with infected animals (6). Among others, poultry products are a prominent source of human salmonellosis, and the contamination can originate from a multitude of sources during poultry production (7–9). *S*. Enteritidis is considered as one of the most commonly identified serovars in association with human infection in the United States (10). The number of human infections by *S*. Enteritidis continued to increase from the 1980s and had reached the point where *S*. Enteritidis became the predominant serovar in the 1990s and currently still remains a prominent foodborne disease-causing serovar (11, 12). Therefore, it is critical not only to understand the transmission modes of *S*. Enteritidis in chicken flocks but also to be able to quantitate their relative contribution of each route to contamination during poultry production. Knowing the quantitative contribution of various transmission routes would be very helpful in designing optimal strategies to minimize the spread of *Salmonella* within a chicken flock *via* interventions such as vaccines and antimicrobials administered in the feed or drinking water (13, 14).

The transmission of *Salmonella* in a chicken flock involves an initial infection with single or multiple *Salmonella* strains from different sources through oral or tracheal routes (15–19). While the oral route is believed to be the primary infection route of *Salmonella* based on experimental evidence (1, 2), there are indications that airborne transmission is also a possible route (20–23). Once infection occurs, the *Salmonella* population disseminates in the host from the entry site and may colonize the intestinal tract or systemically invade the host tissues (24). Once a host becomes infected locally in the intestinal tract or systemically, *Salmonella* can, in turn, be disseminated to other susceptible hosts (25).

*Salmonella*, as an enteric pathogen, can be disseminated to poultry flocks through several sources. Drinking water, feed, wildlife or pets, transportation mode, manure, or litter can be vehicles contributing to dissemination of *Salmonella* into poultry (26). Water is an important vehicle and can serve as a reservoir for *Salmonella* dissemination. *Salmonella* possesses the capacity to not only survive in the water for a long period of time but the expression of key virulence factors can also be increased when *Salmonella* is exposed to stressors in a water environment (27). *Salmonella* appears to possess the mechanisms to retain viability and successfully survive in river environments as well. The relationship between the contaminated feed and the occurrence of *Salmonella* in poultry has been substantiated by several studies (28, 29). For the reason of labor and technical simplification, most chicken feed is produced in the farm as milled and blended mash, most of which are not heat treated or pelleted. The vertical integration nature of the commercial poultry production cycle could impact the risk of introducing pathogens such as *Salmonella* to poultry production as a result of contaminated feed (30, 31).

Quantitative resolution of critical routes for *Salmonella* establishment in chickens requires the ability to track the strains introduced to the flock distinctively using some sort of recoverable signature. Traditionally, *Salmonella* monitoring has been based on techniques such as introducing foreign elements into the candidate strain to construct marker strains that are antibiotic resistant or express genes for fluorescence proteins (32–34). However, in these methods, the risk in introducing phenotypic features into the resulting marker strains is that it could alter the pathogenicity and physiological status such that the resulting strains no longer behave in exactly same fashion as the corresponding wild type. For example, green fluorescence proteins have been shown to alter growth physiology, while exposure to nalidixic acid can influence gene expression (35, 36). It is well established that acquisition of antibiotic resistance often entails fitness cost or enhanced fitness of the pathogenic strains in the absence of selection pressure (37).

The objective of the present study was to evaluate the proof of concept of barcode-tagged isogenic strains of *Salmonella* Enteritidis in broiler chickens using different routes of infection. A series of isogenic *S*. Enteritidis strains in which distinct DNA barcodes were inserted in a functionally neutral locus in the genome were constructed and the resulting strains employed to quantitatively track the transmission routes of the respective strains by profiling the barcode regions using high-throughput sequencing. The advantages of these barcode-tagged strains over previously used marker strains are that each strain can be tracked quantitatively as a distinguishable part of the entire population at high accuracy, allowing for differentiation among multiple barcode-tagged strains as well as discrimination from the environmental *Salmonella* without altering phenotypes or behaviors during infection, colonization, and dissemination.

## Materials and Methods

### Bacterial Strains and Culture Condition

*Salmonella enterica* serovar Enteritidis phage type 13A strain, which is a primary poultry isolate, was originally obtained from the USDA National Veterinary Services Laboratory (Ames, IA, USA). The plasmid pKD4 was used as a template to amplify the kanamycin resistance gene for construction of the barcode-tagged strains. The *Escherichia coli* strain BW25141 carrying pKD4 was inoculated in Luria-Bertani (LB) broth overnight, and plasmid pKD4 was extracted with the illustra plasmidPrep Mini Spin Kit (GE Healthcare Life Sciences). The *Salmonella* Enteritidis strain (SE) containing pKD46 that encodes Red recombinase system was used for construction of barcode-tagged strains *via* electroporation (38, 39). The plasmid pKD46 contains an ampicillin resistance gene and is also a temperature-sensitive replicon requiring 30°C for replication of the plasmid in the cell. LB broth was used for cultivation of barcode-tagged strains. Super optimal broth with catabolite repression (SOC) media (Invitrogen, Carlsbad, CA, USA) was used for phenotypic expression of the transformed cells immediately after the electroporation. Appropriate antibiotics were used at the following concentrations when necessary: kanamycin (Km) at 50 µg/ml and ampicillin (Amp) at 100 µg/ml.

### Rationale for the Genomic Location Selection

**Figure 1:**
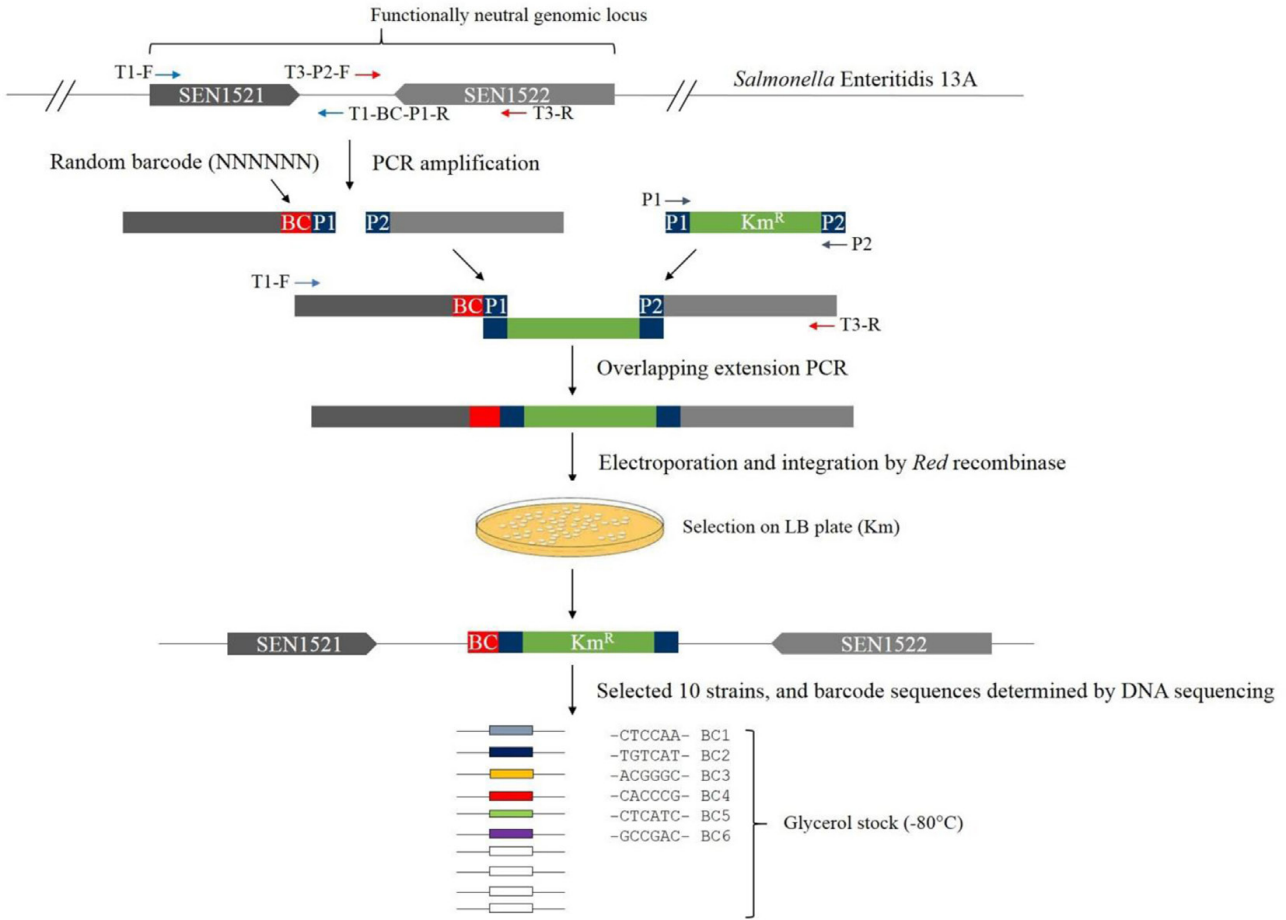
**Schematics of the construction of barcode-tagged *Salmonella* Enteritidis 13A strains**.

Ideally, the barcode along with the kanamycin resistance gene should be inserted into a functionally neutral genomic locus. Based on Chaudhuri et al. (40), we first searched for two adjacent genes that are not required for intestinal colonization in chickens and are also transcribed toward each other. We manually searched for the target locus for barcode insertion in the genome based on the result of Chaudhuri et al. (40) and found that SEN1521 and SEN1522 met these two conditions, and therefore, the intergenic region (141 bp) between these two genes was selected for insertion of a barcode plus the kanamycin resistance gene among other candidate loci (Figure 1). When foreign sequences are inserted in the middle of this intergenic region without removing any original genomic sequences, it can be ensured that the insertion would not cause any polar effect on the downstream genes that would minimize, if any, phenotypic change due to the barcode insertion.

### Construction of Barcode-Tagged Strains

All PCR primers are listed in Table 1. The 3′ end and downstream regions of the coding genes SEN1521 (232 bp) and SE1522 (267 bp) were amplified from the genomic DNA of *S*. Enteritidis 13A with the primer pairs of T1-F and T1-BC (Barcode)-P1-R, and T3-P2-F and T3-R, respectively (termed, upstream and downstream fragments, respectively) (Figure 1). The T1-BC-P1-R primer contained a barcode of six random nucleotides and the sequence overlapping with 5′ end of the Km resistance gene (P1). The T3-P2-F primer contained the sequence overlapping with 3′ end of the Km gene (P2). The Km resistance gene (1,496 bp) was amplified from the plasmid pKD4 with the primer pair of P1 and P2. The PCR assays were conducted by combining approximately 0.1 µg of purified genomic DNA or plasmid along with 1 µl of 2.5 U/μl Pfu polymerase (Agilent Technologies), 5 µl of 10 × cloned Pfu polymerase buffer, 4 µl of 2.5 mM dNTPs (TaKaRa), and 1 µl of 1.2 µM of each primer resulting in a total volume of 50 µl. The DNA Engine^®^ Thermal Cycler (Bio-Rad, Hercules, CA, USA) was used with the following amplification cycles: 94°C for 2 min; 30 cycles of 94°C for 30 s, 58°C for 60 s, 72°C for 60 s per 1 kb; and 72°C for 10 min for the final extension. Each PCR product was gel purified and eluted in 25 µl EB buffer (10 mM Tris–Cl; pH 8.5) for preparation of templates to be used for overlapping extension PCR. Overlapping extension PCR was employed to join the three fragments (upstream fragment plus a barcode + Km resistance gene + downstream fragment) together with the primers T1-F and T3-R (Figure 1). After running the agarose gel for confirmation of the correct size, electroporation was used to introduce the overlapping PCR fragments into *S*. Enteritidis carrying pKD46 plasmid. A number of transformants selected on LB agar plates supplemented with Km were first analyzed by PCR for the presence of the barcode plus kanamycin resistance gene in the correct genomic locus with the primers BC-F and BC-R and, if positive, analyzed for barcode sequences by Sanger sequencing of the PCR products (41). Finally, we isolated and confirmed 10 barcode-tagged strains carrying unique barcodes. Six of them were used in this study, and the barcodes in the respective strains were BC1 (CTCCAA), BC2 (TGTCAT), BC3 (ACGGGC), BC4 (CACCCG), BC5 (CTCATC), and BC6 (GCCGAC).

**Table 1:** **Oligonucleotides used in this study**.

Primers for construction of barcode-tagged strains (5′→3′)	
T1-F	GCAAGGTTGGTGTCTGTCCT
T1-BC-P1-R	GAAGCAGCTCCAGCCTACACNNNNNNATTATTGTTAATTTATTCTT
P1	GTGTAGGCTGGAGCTGCTTC
P2	ATGGGAATTAGCCATGGTCC
T3-P2-F	GGACCATGGCTAATTCCCATAAAGGTTAAGCAGTGACCCA
T3-R	GTTGATGGACTGGGTTCGTT
BC-F	AGCGTCCTGAAATAATAAAAGAA
BC-R	CGGACTGGCTTTCTACGTGT
**Illumina index forward primers (5′→3′) 6 nt-index sequences are underlined**	
AATGATACGGCGACCACCGAGATCTACACTCTTTCCCTACACGACGCTCTTCCGATCTATCACGGCGTCCTGAAATAATAAAAGAATAAA	
AATGATACGGCGACCACCGAGATCTACACTCTTTCCCTACACGACGCTCTTCCGATCTCGATGTGCGTCCTGAAATAATAAAAGAATAAA	
AATGATACGGCGACCACCGAGATCTACACTCTTTCCCTACACGACGCTCTTCCGATCTTTAGGCGCGTCCTGAAATAATAAAAGAATAAA	
AATGATACGGCGACCACCGAGATCTACACTCTTTCCCTACACGACGCTCTTCCGATCTTGACCAGCGTCCTGAAATAATAAAAGAATAAA	
AATGATACGGCGACCACCGAGATCTACACTCTTTCCCTACACGACGCTCTTCCGATCTACATGTGCGTCCTGAAATAATAAAAGAATAAA	
AATGATACGGCGACCACCGAGATCTACACTCTTTCCCTACACGACGCTCTTCCGATCTGCCAATGCGTCCTGAAATAATAAAAGAATAAA	
**Illumina index reverse primers (5′→3′) 6 nt-index sequences are underlined**	
CAAGCAGAAGACGGCATACGAGCTCTTCCGATCTATCACGGAAGCAGCTCCAGCCTACAC	
CAAGCAGAAGACGGCATACGAGCTCTTCCGATCTCGATGTGAAGCAGCTCCAGCCTACAC	
CAAGCAGAAGACGGCATACGAGCTCTTCCGATCTTTAGGCGAAGCAGCTCCAGCCTACAC	
CAAGCAGAAGACGGCATACGAGCTCTTCCGATCTTGACCAGAAGCAGCTCCAGCCTACAC	
CAAGCAGAAGACGGCATACGAGCTCTTCCGATCTACATGTGAAGCAGCTCCAGCCTACAC	
CAAGCAGAAGACGGCATACGAGCTCTTCCGATCTGCCAATGAAGCAGCTCCAGCCTACAC	
CAAGCAGAAGACGGCATACGAGCTCTTCCGATCTCAGATCGAAGCAGCTCCAGCCTACAC	
CAAGCAGAAGACGGCATACGAGCTCTTCCGATCTACTTGAGAAGCAGCTCCAGCCTACAC	
CAAGCAGAAGACGGCATACGAGCTCTTCCGATCTGATCAGGAAGCAGCTCCAGCCTACAC	

*Underline sequences indicate Illumina index adapter*.

### Chicken Infection Experiments

All animal procedures in this study were conducted in accordance with the protocol approved by the University of Arkansas Institutional Animal Care and Use Committee. In all experiments, day-of-hatch broiler chicks were obtained from Cobb-Vantress (Siloam Springs, AR, USA). To test the utility of the barcode-tagged strains for quantitative tracking of *Salmonella* transmission, we set up three independent experiments as described below.

### Oral Infection into Seeder Chickens Experiment 1

Six chickens were randomly selected for oral infection with *Salmonella* barcode-tagged strains (referred to as seeder chickens hereafter) on day 1. Three of the chickens (seeder chickens #1–3) were orally infected at low dose (10^3^ CFUs) with BC1, BC2, and BC3 strains, respectively. The other three chickens (seeder chickens #4–6) were orally infected at high dose (10^5^ CFUs) with BC4, BC5, and BC6 strains, respectively. The other 10 chickens were not infected with any barcode-tagged strains and were referred to as contact chickens (# 7–16). Seeder and contact chickens were housed together for 14 days. On day 7, post-infection three contact chickens (# 7–9) were euthanized, and cecal contents were removed and stored at −20°C for genomic DNA isolation. Each bird foot was washed thoroughly in 5 ml of PBS buffer in a sterile Ziploc bag, and bacterial cells from the rinse were subsequently harvested *via* centrifugation at 4,468 × *g* for 10 min. The bacterial pellets were stored at −20°C and used for genomic DNA isolation. On day 14, four seeder chickens (chick 1, 4, 5, 6; chick #2 and 3 were not sampled) and four contact chicks (chick 10–13) were also euthanized and cecal contents as well as foot wash were collected for DNA isolation as described previously.

### Consumption of Contaminated Feed Experiment 2

The same six barcode-tagged strains were used to inoculate a balanced antibiotic-free corn/soybean-based diet at two different levels: at low dose (10^3^ CFUs) with BC1, BC2, and BC3 strains, respectively, and at high dose (10^5^ CFUs) with BC4, BC5, and BC6 strains, respectively. To minimize the volume of the liquid inoculum, the cell suspension of each barcode-tagged strain was concentrated to contain the target cell number in 1 µl inoculum. We spotted 1 µl of inoculum for each of six barcode-tagged strains on the surface of the feed (1.36 kg) placed in the feeder using a pipette and left it without any mixing to simulate the way *Salmonella* would contaminate feed in the real situation. Sixteen chickens were allowed to consume this contaminated feed for 48 h. After 2 days, the contaminated feed was replaced by *Salmonella*-free feed and water *ad libitum*. On day 7 and 14, two and four chickens were euthanized, respectively. For each euthanized bird, both ceca and foot-wash samples were collected and processed by the same procedures described previously.

### Drinking Water Administration Experiment 3

This experiment was setup essentially in the same way as Experiment 2, except that the six barcode-tagged strains were added to and mixed in 11.36 l of drinking water. Chickens (*n* = 16) were allowed to drink *ad libitum* this contaminated water for 48 h. After 2 days, the contaminated water was replaced with *Salmonella*-free fresh water. On days 7 and 14, four chicks were euthanized, respectively. Cecal and foot-wash samples were collected and processed by the same procedure described previously.

### Illumina Sequence Sample Preparation

Genomic DNA was isolated from each sample using QIAamp DNA MiniKit (Qiagen). The concentration of purified DNA was measured by a Qubit^®^3.0 Fluorometer (ThermoFisher Scientific). Subsequently, the barcode regions in the extracted genomic DNA of each sample were amplified using the primers BC-F and BC-R (Table 1), and G2 PCR mixture (Promega) with an initial incubation of 2 min at 95°C followed by 35 cycles of 30 s at 94°C, 1 min at 55°C, and 1 min at 72°C followed by a 10 min extension at 72°C. The PCR products of 191 bp were purified by using a QIAquick PCR purification kit (Qiagen) for use as a template in the next round of PCR. The second step PCR was conducted to attach Illumina-specific sequences along with the combinatorial sample index sequences (6 nt) on both ends using the Illumina index forward and reverse primers shown in Table 1. A total of nine Illumina index forward and six Illumina index reverse primers were used, allowing up to 54 (9 × 6) samples to be sequenced simultaneously. The resulting amplicons of 167 bp were purified by ethanol purification method and were pooled together to generate an amplicon library for MiSeq sequencing with single-end read option *via* 150 cycles.

### Analysis of DNA Sequencing Results

Custom Perl script was used to perform the following data analysis: first, the barcode regions of 57 bp in the sequence reads from Illumina MiSeq data were extracted. The 12 bp-index sequences were obtained by extracting and combining forward index sequence (6 bp) and reverse index sequence (6 bp) and used to sort the barcode reads to different samples. The six different barcodes were subsequently extracted and used to determine the relative abundance of different barcode-tagged strains in each sample.

## Results and Discussion

### Quantitative Profiling of Barcode-Tagged Strains

A total of 1,461,014 sequence reads of 150 bp were obtained from the MiSeq sequencing run. The sequence reads were binned into different files according to the combinatorial index sequences corresponding to the samples from the three experiments. If any reads did not match perfectly to one of the original six barcode sequences, they were subsequently deleted. Since the read numbers reflect only relative frequency of each barcode-tagged strain in a given sample, the original read numbers were converted to calculate the percentage of each barcode-tagged strain in each sample.

### Experiment 1: *Salmonella* Transmission after Oral Infection

The results of transmission of the SE barcode-tagged strains in the cecal content and foot wash of seeder chickens on day 14 from Experiment 1 are summarized in Table 2. For cecal samples of seeder bird #1, which was infected with BC1 strain at low dose (10^3^ CFUs), the BC1 strain was the predominant colonizer (46.37%); however, the other strains challenged at a higher dose (10^5^ CFUs) were also recovered from cecal content of chicken 1: BC3 (20.40%), BC4 (29.63%), and BC5 (3.59%). These results suggest that a significant mixed infection by different *S*. Enteritidis BC strains could occur when the chick was infected by barcode-tagged strains at low dose and subsequently comingled with other infected chickens. The barcode-tagged strains used in this study are isogenic strains with the identical genome sequence except for the barcode region. Therefore, it is possible that the multiple barcode-tagged strains may be recognized as the same strains from each other and/or by the host, leading to avoidance of the exclusion mechanism(s) observed among different strains as has been described previously in chickens and mammals (42–44). In the seeder chickens #4, #5, and #6 infected by respective barcode-tagged strains at high dose, the barcode-tagged strains used for infection were the dominant strains (93.21, 98.56, and 99.94%, respectively) in the ceca (Table 2). It appears that barcode-tagged strains introduced at high dose saturated all potential colonization niches, thus impeding colonization by other strains. This phenomenon observed in the chicks infected by a high dose of *Salmonella* is consistent with the colonization inhibition theory (42, 43). In conclusion, these results suggest that the outcome of cecal colonization in terms of the number of barcode-tagged strains colonizing the ceca is dose dependent, and a high dose beyond a certain threshold level results in dominant colonization by a single strain.

**Table 2:** **Relative abundance of the *Salmonella* Enteritidis (SE) barcode-tagged strains in seeder chickens on day 14 in the cecal content and foot wash from Experiment 1**.

	BC1	BC2	BC3	BC4	BC5	BC6
**Ceca content**						
Chicken 1	46.37%	0.00%	20.40%	29.63%	3.59%	0.01%
BC1/10^3^						
Chicken 4	0.04%	0.004%	0.00%	93.21%	6.74%	0.01%
BC4/10^5^						
Chicken 5	0.04%	0.01%	0.27%	0.00%	98.56%	1.12%
BC5/10^5^						
Chicken 6	0.03%	0.00%	0.004%	0.00%	0.03%	99.94%
BC6/10^5^						
**Foot wash**						
Chicken 1	0.03%	6.37%	0.00%	0.00%	0.02%	93.57%
BC1/10^3^						
Chicken 4	0.42%	0.01%	0.00%	0.01%	99.55%	0.00%
BC4/10^5^						
Chicken 5	0.04%	0.003%	0.00%	36.77%	52.97%	10.21%
BC5/10^5^						
Chicken 6	0.03%	0.005%	0.00%	0.002%	21.10%	78.86%
BC6/10^5^						

*Six chickens were randomly selected for oral infection with *Salmonella* barcode-tagged strains on day 1. Chickens 1 through 3 were orally infected with 10^3^ CFUs with BC1, BC2, and BC3 strains, respectively. Chickens 4 through 6 were orally infected with 10^5^ CFUs with BC4, BC5, and BC6 strains, respectively. At 14 days post challenge, cecal content or foot wash sample was collected from each chicken and used for isolation of genomic DNA. Following PCR and MiSeq analyses of barcode regions, the number of the sequence reads corresponding to different barcodes were used to determine the relative abundance (%) of each SE barcode strain from each chick. Chickens 2 and 3 orally gavaged with BC 2 and BC 3 were not sampled in this experiment*.

Contamination of feet by dominant barcode-tagged strains occurred for the seeder chickens #1, #4, and #6 (93.57% of BC6, 99.55% of BC5, and 78.86% of BC6, respectively), but they were not necessarily the same strains used for infection of the same chickens (Table 2). In the case of seeder bird #5, the foot was contaminated by three strains, BC4, BC5, and BC6 strains (36.77, 52.97, and 21.10%, respectively) among which BC5 was the one used for oral infection of the bird. The vast majority of the strains contaminating feet were those used for infection at high dose (BC4, BC5, and BC6), which indicated that high dose of *Salmonella* BCs is widely disseminated in the environment and thus may frequently be isolated from the feet. However, there is no correlation between the orally infected strain and dominant strain occurring on the feet. It is possible that the major strain isolated from the feet is from the environment instead of coming from chick itself.

**Figure 2:**
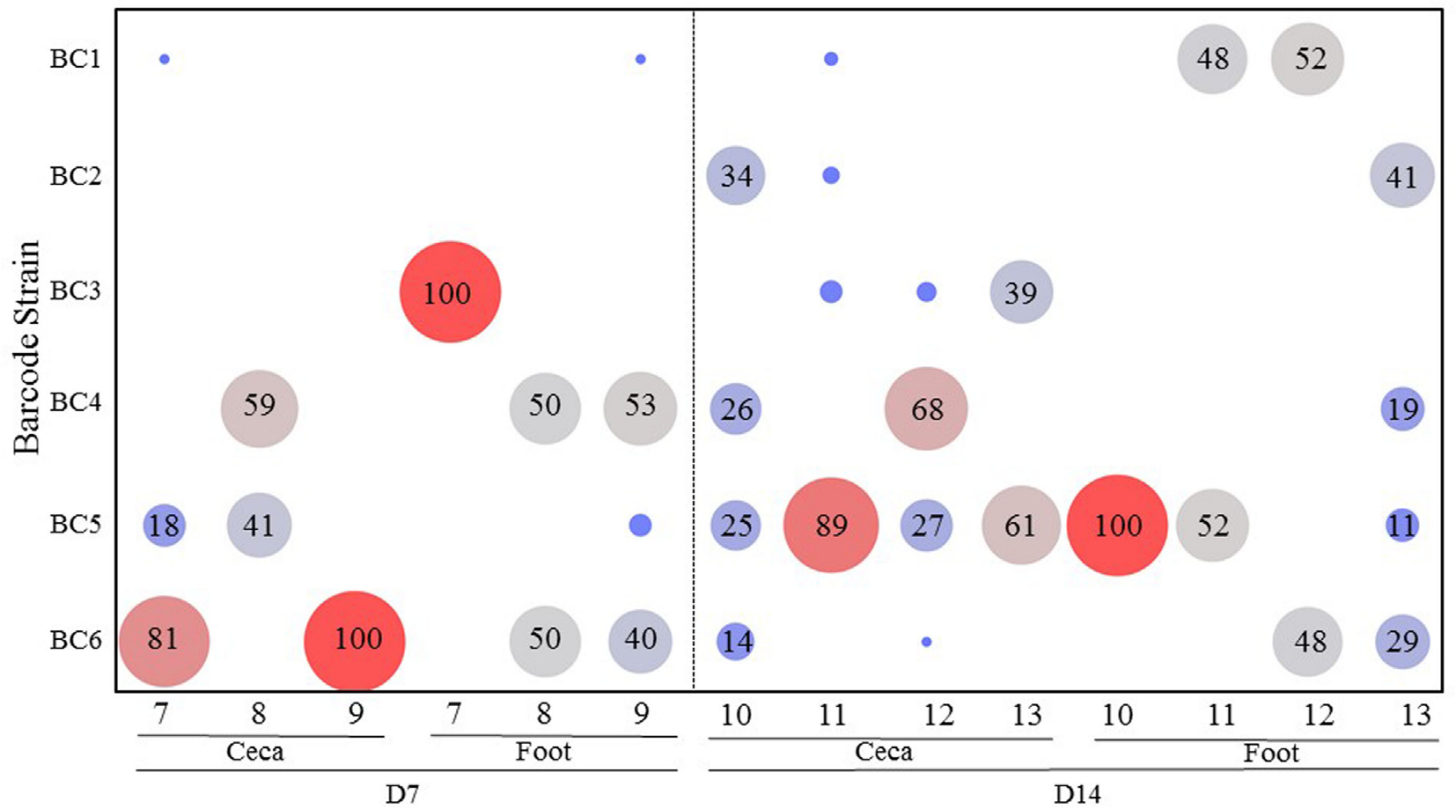
**Transmission of the *Salmonella* barcode-tagged strains in contact chickens in oral infection model**. In Experiment 1, six seeder chickens were infected by different dose of SE barcode strains (BC1, BC2, and BC3 are used for infection of three chickens at 10^3^ CFUs; BC4, BC5, and BC6 are used for infection of other three chickens at 10^5^ CFUs). Other 10 chickens were roomed together with these six seeder chickens and named as contact chickens. Three contact chickens were euthanized on day 7, and four contact chickens were euthanized on day 14. The cecal tonsil and foot wash samples were collected from each chicken by aseptic technique. *x*-axis represents different contact chickens from Experiment 1, and *y*-axis represents different SE barcode strains. The number in bubble presents the relative abundance of each barcode strain in each chicken. Bigger size and red color means the higher relative abundance, and smaller size and blue color means lower relative abundance.

Figure 2 shows the results of transmission of the *S*. Enteritidis barcode-tagged strains in the oral infection model in contact chickens. For the contact chickens, almost all (99%) of the barcode-tagged strains colonizing ceca on day 7 were strains administered at high dose, namely BC4-6. However, on day 14, a more diverse set of barcode-tagged strains were detected from the ceca of contact chickens, including a greater proportion of the barcode-tagged strains that were used to infect seeder chickens at low dose (BC1-3). It seems that the contact chickens are more likely to be colonized by the strains initially used for infection at high dose, but they eventually become colonized in the ceca also by the strains originating from the low dose as time progresses (Figure 2). In contrast, foot-wash samples from all contact chickens did not reveal any obvious trends as compared to those observed in cecal samples. On day 7, BC3 strain, which was administered at low dose, was the only strain (100%) contaminating the foot of the contact bird #7. Conversely, the feet of the contact chickens #8 and 9 were colonized mainly by the two strains, BC4 and BC6, which were used for infection at high dose. After the passage of time, the barcode-tagged strain populations on the feet of the contact chickens became more diverse on day 14. Comparing the relative abundance between days 7 and 14 indicated that the barcode-tagged strains that were used for infection at low dose increased the chances to contaminate the feet with the exception of BC3, which was not detected on the feet of any bird on day 14.

### Experiment 2: *Salmonella* Transmission after Infection through Contaminated Feed

**Figure 3:**
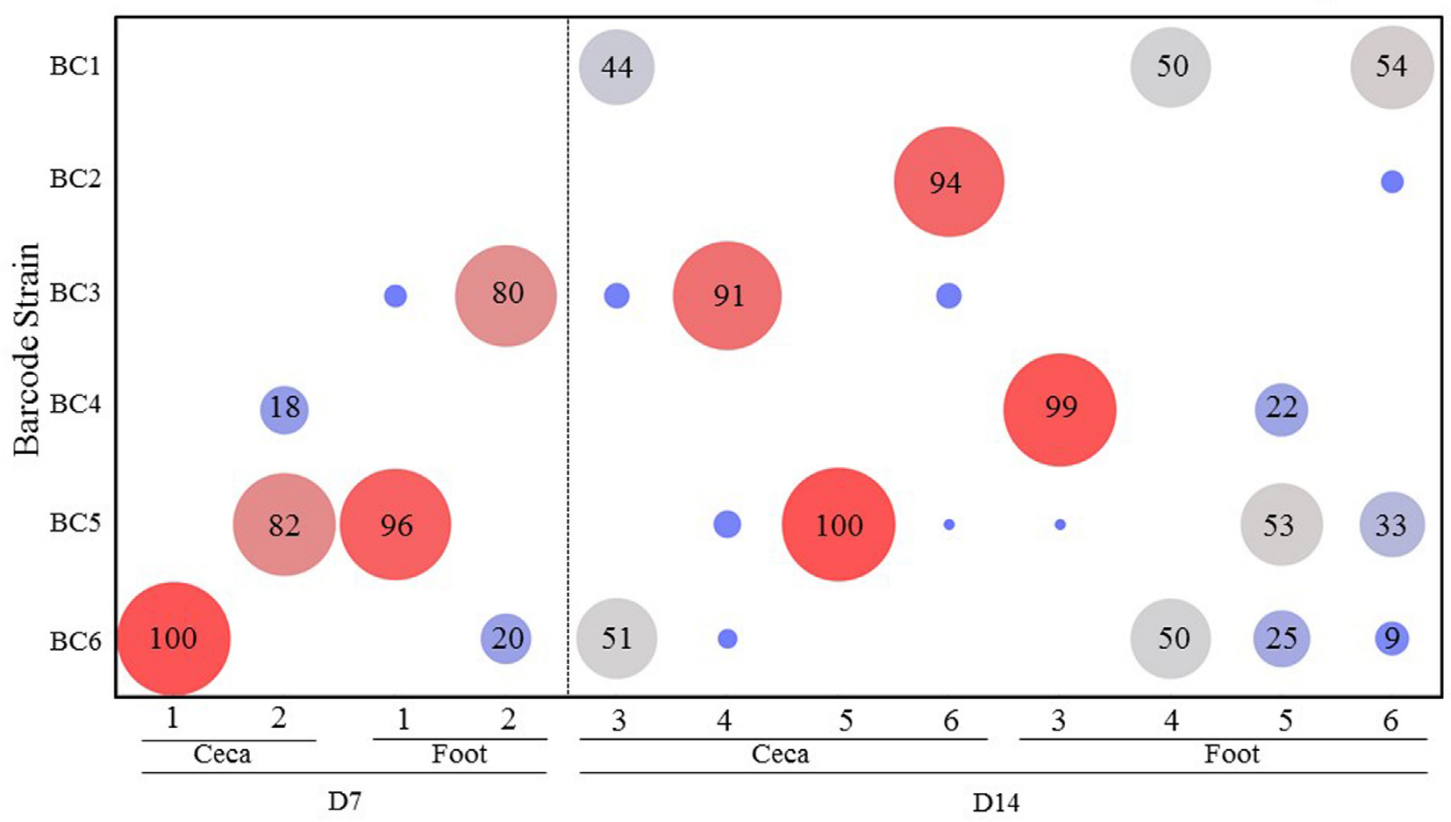
**Transmission of the *Salmonella* barcode-tagged strains in feed contamination model**. In Experiment 2, the feed was contaminated by six SE barcode strains at two doses (BC1, BC2, and BC3 were introduced into the feed at 10^3^ CFUs, and BC4, BC5, and BC6 were at 10^5^ CFUs on day 1). Two chickens were euthanized on day 7, and the other four chickens were euthanized on day 14. The cecal and foot wash samples were collected from each chicken by aseptic technique and used for isolation of genomic DNA. Following PCR and MiSeq analyses of barcode regions, the number of sequence reads corresponding to different barcodes were used to determine the relative abundance (%) of each SE barcode strain from each sample. *x*-axis represents different chickens from Experiment 2, and *y*-axis represents different SE barcode strains. Bigger size and red color means the higher relative abundance, and smaller size and blue color means lower relative abundance.

The results of transmission of the SE barcode-tagged strains in a feed contamination model (Experiment 2) are shown in Figure 3. On day 7, the ceca from the two chickens were colonized mainly by the barcode-tagged strains that were introduced at the higher dose. On day 14, the ceca from the birds #4 and 6 were predominantly colonized by BC3 (91%) and BC2 (94%) (both introduced at a low dose), respectively, while bird #5 was exclusively colonized by BC5. On day 14, only bird #3 showed colonization by multiple strains, mostly BC1 (44%) and BC6 (51%) strains. By comparing the combined percentages of the low versus high dose strains in the ceca at day 7 (0 versus 100%) and day 14 (60 versus 40%), it is apparent that the strains introduced to feed at a low dose eventually colonized the ceca, but it required a much longer period of time when compared to the strains introduced at high dose. Greater diversity of the strains was also detected at day 14 as compared to day 7 for the feet samples with the exception of bird #3 (Figure 3).

### Experiment 3: *Salmonella* Transmission after Infection through Contaminated Drinking Water

**Figure 4:**
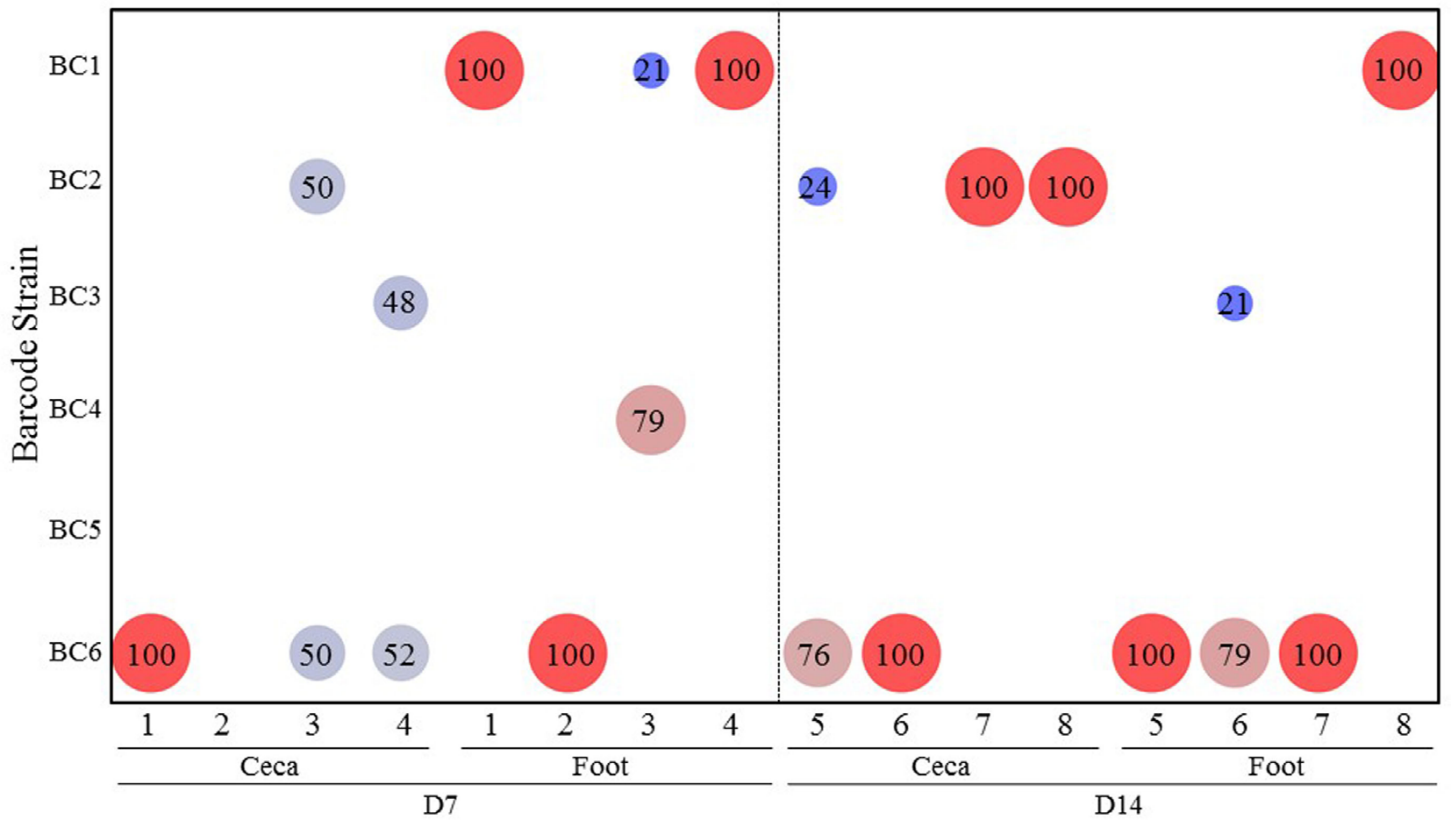
**Transmission of the *Salmonella* barcode-tagged strains in water contamination model**. In Experiment 3, the water was contaminated by 6 SE barcode strains at two doses (BC1, BC2, and BC3 were introduced into the water at 10^3^ CFUs, and BC4, BC5, and BC6 were at 10^5^ CFUs on day 1). Four chickens were euthanized on day 7, and the other four chickens were euthanized on day 14. The cecal and foot wash samples were collected from each chicken by aseptic technique and used for isolation of genomic DNA. Following PCR and MiSeq analyses of barcode regions, the number of sequence reads corresponding to different barcodes were used to determine the relative abundance (%) of each SE barcode strain from each sample. *x*-axis represents different chickens from Experiment 2, and *y*-axis represents different SE barcode strains. Bigger size and red color means the higher relative abundance, and smaller size and blue color means lower relative abundance.

Figure 4 summarizes the results of transmission of the SE barcode-tagged strains in water contamination model (Experiment 3). When the chicks were infected through contaminated drinking water, only three barcode-tagged strains (BC2, BC3, and BC6), representing both the strains that had been introduced at low and high dose, were recovered from the ceca on days 7 and 14. Strain BC6 (high dose), which was the predominant cecal colonizer, was also detected as the predominant strain contaminating the feet. Interestingly, BC1 (a low-dose challenge strain), even though it was not detected in the ceca of any chicken at any time, was recovered as the predominant strain in the feet of the chickens (Figure 4). Since only 8 chickens were analyzed out of the total of 16 chickens, BC1 is the predominant colonizer in at least one of the remaining chickens that was not used for sample collection.

## Conclusion

*Salmonella* transmission in chicken flocks has already been the subject of several studies in which the *Salmonella* strains introduced to the flock were identified and quantified by culturing on selective agar plates and confirmed by biochemical and serological methods (45–50). In the studies conducted by De Vylder et al. (47) and Thomas et al. (48, 49), single *Salmonella* Enteritidis strains were used to analyze different aspects of *Salmonella* transmission within the laying hen flocks. These approaches have been useful in understanding the impact of different phage type strains or housing system on the frequency of horizontal transmission (47, 50, 51) or measuring different parameters of *Salmonella* transmission (48). However, a detailed picture of transmission involving interactions among multiple strains or serotypes cannot be investigated using the culture methods, due to the inability to differentiate multiple strains based on the culture methods.

Several investigators have studied the persistence of horizontal fecal shedding of *Salmonella* Enteritidis in experimentally infected laying hens housed on different commercial conditions (50, 51). However, these studies are still limited to reflect the complexity of the environmental conditions that *Salmonella* is exposed to during transmission in a poultry farm. The other weakness of culture method approaches is that the isolated strains may be from environment rather than the strain externally introduced as a part of an experimental infection, thus handicapping the ability to differentiate the corresponding strain. Even though the strain might be confirmed as an experimental strain by further characterization, the result can only indicate the presence of the strain and reliable quantification is not possible.

In order to quantitatively track the *Salmonella* transmission routes from environment to flock, we constructed a series of barcode-tagged strains, which carry distinct barcode tags that would allow them to be identified and quantified accurately by high-throughput sequencing of the barcode regions. Similar methods of barcode tagging have been applied to understand the transmission dynamics within the infected hosts for *Salmonella* (52, 53), other pathogenic bacteria (54), and viruses (55). However, to our knowledge, this is the first report on the application of the barcode-tagged strains to study transmission dynamics within a population of the host animals. In this study, we used the barcode-tagged strains of *S*. Enteritidis to understand the transmission dynamics of *Salmonella* in a quantitative manner after initial introduction through oral infection or consumption of contaminated feed or drinking water.

In the current study, six barcode-tagged *S*. Enteritidis strains were employed to infect six chickens (seeder chickens) orally in oral infection experiment. In contaminated feed and water study, the same six barcode-tagged strains were introduced into feed or water in each isolator. Following the exposure *via* different routes, the corresponding distributions of the six different barcode-tagged strains at different colonization sites (ceca and feet) were analyzed at different time points post-infection.

Utilizing PCR and Illumina MiSeq analyses, the population structure could be assessed and representative transmission figures could be constructed. The results are important for understanding the patterns of *S*. Enteritidis dissemination in poultry and are revealed by demonstrating that a higher dose of *S*. Enteritidis has a greater opportunity to infect flocks. In addition, the data from this study suggest that colonization-inhibition by competing *Salmonella* is somewhat dosage dependent. Based on qPCR result for quantification of the combined load of all barcode-tagged strains (data not shown), it appears that recovery of *S*. Enteritidis barcode-tagged strains introduced orally were not different among the seeder chickens and contact chicks in both cecal and foot-wash samples on day 14. All barcode strains combined in the cecal samples remained stable on days 7 and 14 in Experiment 1, while those from foot-wash samples increased 10-fold in the three experiments after time had elapsed.

To better establish the implications for commercial poultry production settings, larger scale experiments are needed to assess additional environmental and host factors. However, the current experiment demonstrated the proof of concept that the use of barcode-tagged strains is a novel and an effective approach to understand the dynamics of *Salmonella* transmission within a chicken flock and can provide valuable insights for the potential to develop and optimize measures that protect host animals from infection with *Salmonella*. Studies to evaluate and confirm previous work published by our laboratory (18–20, 24) that demonstrate the importance of airborne transmission of *Salmonella* versus oral infection as well as the competitive exclusion concept of *Salmonella* versus *Salmonella* (43, 56) or cross protection (44, 57, 58) using these SE barcode-tagged strains are currently in progress.

## Author Contributions

YK, YY, SR, and GT edited and wrote the manuscript and edited the drafts.

## Conflict of Interest Statement

The authors declare that the research was conducted in the absence of any commercial or financial relationships that could be construed as a potential conflict of interest.
